# Docosahexaenoic Acid and Melatonin Prevent Impaired Oligodendrogenesis Induced by Intrauterine Growth Restriction (IUGR)

**DOI:** 10.3390/biomedicines10051205

**Published:** 2022-05-23

**Authors:** Britta Anna Kühne, Paula Vázquez-Aristizabal, Mercè Fuentes-Amell, Laura Pla, Carla Loreiro, Jesús Gómez-Catalán, Eduard Gratacós, Miriam Illa, Marta Barenys

**Affiliations:** 1 Grup de Recerca en Toxicologia (GRET), INSA-UB and Toxicology Unit, Pharmacology, Toxicology and Therapeutical Chemistry Department, Faculty of Pharmacy, University of Barcelona, 08028 Barcelona, Spain; brkuhnek24@alumnes.ub.edu (B.A.K.); vazquezaristizabal@gmail.com (P.V.-A.); mfuentam7@alumnes.ub.edu (M.F.-A.); jesusgomez@ub.edu (J.G.-C.); 2 BCNatal | Fetal Medicine Research Center (Hospital Clínic and Hospital Sant Joan de Déu), Universitat de Barcelona, 08028 Barcelona, Spain; laura.pla.codina@gmail.com (L.P.); carla.lr2@gmail.com (C.L.); egratacos@ub.edu (E.G.); miriamil@clinic.cat (M.I.); 3 Institut d’Investigacions Biomèdiques August Pi i Sunyer (IDIBAPS), 08036 Barcelona, Spain; 4 Center for Biomedical Research on Rare Diseases (CIBER-ER), 08036 Barcelona, Spain; 5 Institut de Recerca Sant Joan de Déu, 08950 Esplugues de Llobregat, Spain

**Keywords:** progenitor cells, cell culture, differentiation, oligodendrocytes, nervous system development, neurogenesis, fetal growth restriction

## Abstract

In this study, our aims were to characterize oligodendrogenesis alterations in fetuses with intrauterine growth restriction (IUGR) and to find therapeutic strategies to prevent/treat them using a novel rabbit in vitro neurosphere culture. IUGR was surgically induced in one uterine horn of pregnant rabbits, while the contralateral horn served as a control. Neural progenitor cells (NPCs) were obtained from pup’s whole brain and cultured as neurospheres mimicking the basic processes of brain development including migration and cell differentiation. Five substances, chosen based on evidence provided in the literature, were screened in vitro in neurospheres from untreated rabbits: Docosahexaenoic acid (DHA), melatonin (MEL), zinc, 3,3′,5-Triiodo-L-thyronine (T3), and lactoferrin (LF) or its metabolite sialic acid (SA). DHA, MEL and LF were further selected for in vivo administration and subsequent evaluation in the Neurosphere Assay. In the IUGR culture, we observed a significantly reduced percentage of oligodendrocytes (OLs) which correlated with clinical findings indicating white matter injury in IUGR infants. We identified DHA and MEL as the most effective therapies. In all cases, our in vitro rabbit neurosphere assay predicted the outcome of the in vivo administration of the therapies and confirmed the reliability of the model, making it a powerful and consistent tool to select new neuroprotective therapies.

## 1. Introduction

Brain development is one of the most sensitive and vulnerable processes during pregnancy. Its disturbance can manifest in neurobehavioral disorders throughout life [1]. Intrauterine growth restriction (IUGR) is defined as a pathological fetal condition where the fetus has not attained its biologically determined growth potential and estimated fetal weight is below the 10th percentile for gestational age. IUGR is among the most frequent disorders, affecting 5–10% of all pregnancies [2]. Most commonly, IUGR occurs due to abnormal placental function, which reduces placental blood flow, leading to fetal development under chronic hypoxia. Restricted oxygen and nutrition supply can have serious consequences for the developing brain, disrupting the normal patterns of gray and white matter development [3,4]. White matter injury occurs due to impaired myelination and oligodendrocyte maturation, which lead to adverse neurodevelopmental sequelae [5,6,7,8]. In the long run, IUGR-infants are prone to develop neurocognitive disorders, learning disabilities, attention deficit hyperactivity disorder, or autism spectrum disorder [1,9,10,11]. However, there is currently no therapy to prevent or revert, even at the prenatal period, the neurological insults that arise from IUGR [12,13].

With the aim of testing potential therapies applicable during the prenatal period to prevent IUGR-induced neurological disorders, we recently established an in vitro rabbit neurosphere model reproducing brain development under chronic and mild IUGR conditions [14]. In this model, neurospheres are prepared from a rabbit in vivo model mimicking placental insufficiency by selective ligation of uteroplacental vessels in late pregnancy. The rabbit in vivo model has already been shown to present cardiovascular Doppler changes similar to human IUGR, reduced birth weight and a higher brain to birth weight ratios [4,15,16]. The neurosphere model, prepared from IUGR and control postnatal day 0 (PND0) pups’ whole brains, simulates the basic functions of brain development including cell proliferation, migration and differentiation to neurons, astrocytes, and oligodendrocytes (OLs) [14,17,18]. This 3D in vitro culture is an efficient choice because several potential therapies can be tested cost-effectively and more ethically than in in vivo experiments. In this technique, a wide concentration range of each potential therapy can be tested to select the in vivo concentration range of interest. Afterwards, it is also possible to prepare neurospheres from pups’ brains exposed to the therapies in vivo during gestation to test the efficacy and safety of the treatment within the previously selected concentration range.

By using the neurosphere model, it is also possible to study the effects of nervous system diseases like Alzheimer’s disease or glioma [19] as well as the mode of action of compounds [20,21]. This culture can be used to characterize the effects at a cellular level to fill the gaps in translational approaches going from in vitro functional alterations to in vivo, known adverse outcomes. With the rabbit neurosphere model, we previously discovered that IUGR has a severe impact on OL formation, reducing its differentiation significantly [14] and confirming previous results of impaired OL formation after IUGR in vivo [4,8]. In the present study, we included a time-course evaluation and a molecular characterization of this impact produced by IUGR in OLs. We analyzed the maturational stages over time and the expression of two major genes involved in myelination: Myelin basic protein (*Mbp*) and myelin oligodendrocyte glycoprotein (*Mog*). In parallel, the neurosphere assay included measurements of radial glial migration and cell viability.

Aiming to correct the identified adverse effect of IUGR in OLs, we further tested five potential therapies: docosahexaenoic acid (DHA), melatonin (MEL), zinc, L-triiodothyronine (T3), lactoferrin (LF), and its main metabolite sialic acid (SA). All therapies were selected based on literature research which indicated promising results to overcome fetal neurological disorders (Table S1). DHA, a long-chain polyunsaturated fatty acid, is essential for fetal brain development due to its contribution to myelin formation, neurotransmitter metabolism, and synaptogenesis leading to better maintenance of neuronal networks [22,23,24]. The hormone MEL reportedly reduces fetoplacental oxidative stress and is effective at reducing cerebral white and gray matter injury arising from placental insufficiency and IUGR in vivo in sheep [25]. Zinc is crucial for normal brain development because its deficit harms neuronal migration and differentiation and triggers apoptosis [26,27,28,29]. Thyroid hormones regulate the growth of the fetus and its brain development by supporting especially OL maturation [30,31,32]. LF is a sialic acid-rich glycoprotein that restores IUGR-induced impaired oligodendrocyte precursor cell marker NG2 [33] and supports neuronal outgrowth and synaptic connectivity during fetal brain development [34].

From the five potential therapies tested in vitro, three of them were selected to be administered in vivo during pregnancy after IUGR induction to find the most promising candidates to prevent/revert OL damage associated with IUGR.

## 2. Materials and Methods

### 2.1. In Vivo Procedures: IUGR Induction and Administration of Therapies

**Figure 1:**
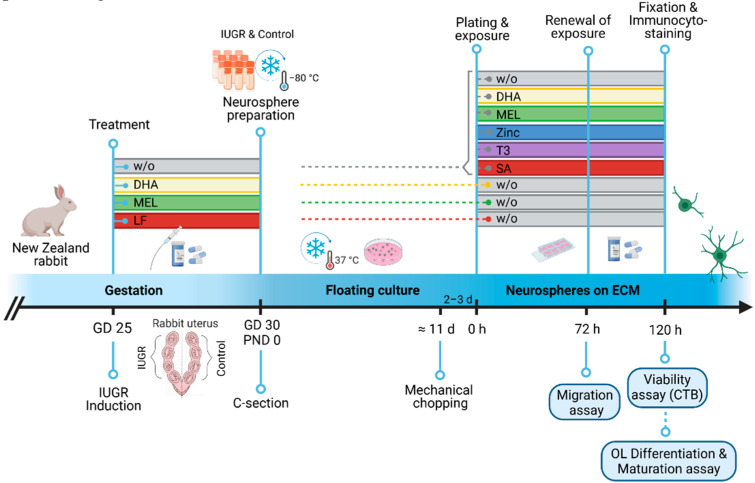
Experimental setup. IUGR was induced in one uterine horn of pregnant rabbits on gestational day (GD) 25, whereas the contralateral horn remained as control. No treatment (w/o) or therapies were administered to the pregnant rabbit until C-section (GD 30). On PND 0, IUGR and control pups were obtained from every group and neurospheres prepared from pup’s whole brain. Neurospheres were cultivated in a floating culture for approx. 11 days and mechanically chopped 2–3 days before plating. On the experimental day (0 h), neurospheres (0.3 mm) were plated on a PDL/Laminin coated eight-chamber slide w/o or with exposure to therapies. After 72 h migration distanced was measured and after 120 h viability, oligodendrocyte (OL) differentiation & maturation assessed. Rectangle bars = time of administration or exposure, blue circle = endpoints. w/o = without, GD = gestational day, PND = postnatal day, ECM = extracellular matrix, OL = oligodendrocyte, CTB = cell titer blue. Created with BioRender.com (accessed on 25 April 2022).

All animal experimentation procedures were approved by the Ethics Committee for Animal Experimentation (CEEA) of the University of Barcelona. All protocols were accepted by the Department of Environment and Housing of the Generalitat de Catalunya with the license number: 11126, date of approval 24 May 2021, and the procedure CEEA number OB 340/19 SJD. The procedure for the IUGR induction was previously described in Eixarch et al., 2009 [15]. Briefly, IUGR was induced at 25th gestational day (GD 25) of pregnant New Zealand rabbits by surgical ligature of 40–50% of the uteroplacental vessels of each gestational sac of one uterine horn (IUGR group), the contralateral horn was left for normal growth (control group). At the time of IUGR induction, pregnant rabbits were randomly assigned to 4 groups: without treatment (w/o), treatment with DHA, MEL or LF (Table 1, Figure 1). The therapies were administered orally to pregnant rabbits on the day of IUGR induction (GD 25) until Caesarean (C-) section was carried out at GD 30 followed by body weight measurement. For all groups, the inclusion criteria of IUGR PND0 rabbit pups was a birth weight lower and for control pups higher than the 25th percentile (39.7 g, Table 1 in Barenys et al., 2021 [14]). The in vitro neurosphere culture was generated by decapitation and whole-brain dissection at PND0 from control and IUGR pups. The administered dose, number, and birth weight of PND0 rabbit pups are listed in Table 1. Information about in vivo treatment calculations and supplier is presented in Supplemental Material 1 (SM1).

### 2.2. Neurosphere Preparation

Rabbit neural progenitor cells (NPCs) were isolated from rabbits’ whole brains. Meninges and olfactory bulbs were discarded followed by mechanical chopping, enzymatic digestion (20 min incubation with papain 20 U/mL at 37 °C), mechanical homogenization into a cell suspension, and centrifugation (10 min at 1200 rpm). The cell pellet obtained was resuspended in 1 mL freezing medium (1:1; volume of pellet: volume of freezing medium [consisting in 70% (*v*/*v*) proliferation medium, 20% (*v*/*v*) fetal calf serum and 10% (*v*/*v*) DMSO]) and immediately stored at −80 °C.

After thawing, the freezing medium was replaced by proliferation medium [consisting of DMEM and Hams F12 3:1 supplemented with 2% B27 (Invitrogen, Madrid, Spain), and 20 ng/mL epidermal growth factor (EGF) including recombinant human fibroblast growth factor (FGF2), 100 U/mL penicillin, and 100 µg/mL streptomycin] supplemented with Rho kinase (ROCK) inhibitor Y-276322 at a final concentration of 10 µM. NPCs were cultured for 11 days on Petri dishes coated with poly-HEMA. Half of the medium was replaced every 2–3 days.

### 2.3. The Neurosphere Assay

Two days before starting the neurosphere assay, neurospheres were mechanically chopped to a size of 0.2 mm (McIlwain tissue chopper) to ensure homogeneous size and spherical shape. On the experimental plating day, neurospheres of 0.3 mm in diameter were selected and transferred to PDL/laminin-coated, eight-chamber slides (Falcon, Madrid, Spain) containing 500 µL differentiation medium [consisting of DMEM and Hams F12 3:1 supplemented with N2 (Invitrogen, Madrid, Spain), penicillin, and streptomycin (100 U/mL and 100 μg/mL)] to assess migration, differentiation, and viability. Five neurospheres were plated in each chamber representing intra-experiment replicates. Subsequently, at least three independent experiments were performed for every endpoint and exposure (Figure 1). 

#### 2.3.1. In Vitro Testing of Potential Therapies

Therapies were dissolved in their corresponding solvent depending on their maximum solubility (Table 2), and subsequently, in differentiation medium. Under differentiation conditions, NPCs were exposed for 5 days to the therapies and the exposure medium was renewed every 2–3 days. These 5 days of exposure were chosen because at this time-point, a significant difference of OL differentiation between control and IUGR cultures was previously detected [14], and because it is a time-point that makes it possible to observe all maturation stages of O4+ cells. Basic processes of neurogenesis were assessed to determine the maximum tolerated concentration (MTC) and most effective concentration (EC). The criteria to define the MTC was a viability >70% of solvent control (SC) values, a not significantly reduced migration distance, or a not significantly reduced OL percentage compared to the SC.

#### 2.3.2. Migration Assay

Five neurospheres per chamber were plated in PDL/laminin-coated eight-chamber slides filled with 500 µL differentiation medium. After 48 or 72 h under differentiation conditions, bright-field pictures were taken to monitor migration progression [EX-H30 camera (Casio, Japan)]. Migration distances were determined by measuring the distance from the sphere core to the furthest migrated cell at four pre-defined positions per neurosphere using ImageJ 1.53a software. For the time-course-assay, migration distance was measured every 24 h over 5 consecutive days. The src kinase inhibitor PP2 at 10 μM served as endpoint-specific positive control in every experiment.

#### 2.3.3. OL Differentiation and Maturation Assay

**Figure 2:**
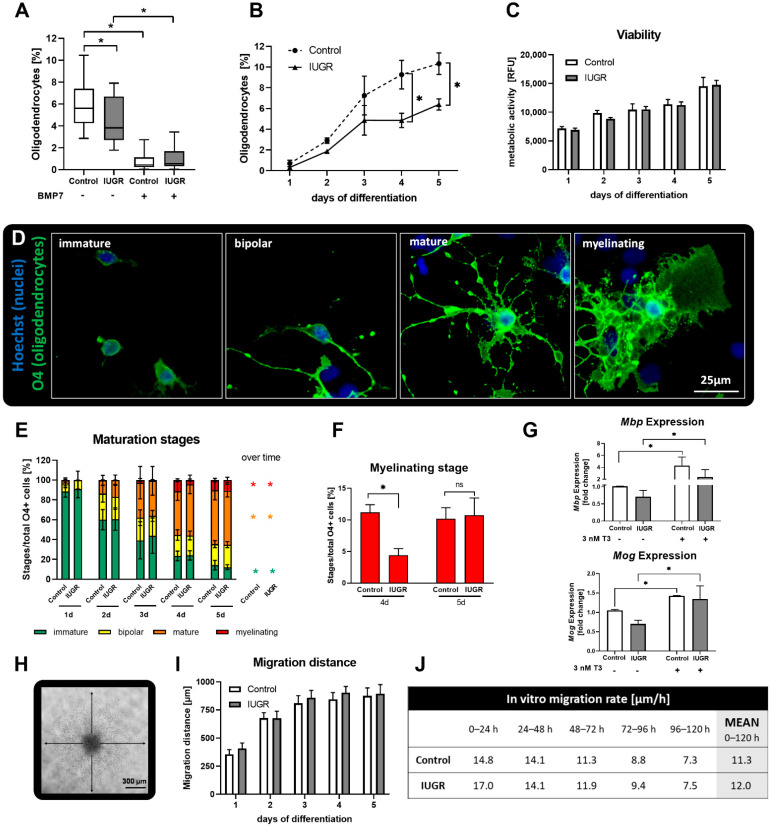
Oligodendrogenesis. Rabbit neurospheres obtained from control and IUGR pups were comparatively analyzed for each endpoint of the ‘Neurosphere Assay’. (**A**) Oligodendrocyte differentiation after 5 days with and without exposure to the positive control BMP7 [100 µM], (**B**) oligodendrocyte differentiation over five consecutive days, (**C**) cell viability determined by metabolic activity, (**D**) representative pictures of maturation stages in control neurospheres, from left to right: immature, bipolar, mature, myelinating with oligodendrocyte marker O4 (green) and nuclei marker Hoechst 33,258 (blue), scale bar = 25 µm. (**E**) Maturation stages of oligodendrocytes (O4+ cells) evaluated by morphological appearance over five days, (**F**) myelinating stage after 4 and 5 days, (**G**) qRT-PCR from *Mbp* and *Mog* expression in control and IUGR neurospheres, with and without exposure to the positive control 3 nM T3. (**H**) Representative picture of migrated NPCs after 3 days, (**I**) migration distance [µm] and (**J**) Migration rate [µm/h]. Mean ± SEM; ns: not significant, *: *p* < 0.05.

After 5 days under differentiation conditions, neurospheres were fixed with 4% PFA for 30 min at 37 °C, washed and stored in PBS until immunostaining. Slides were washed with PBS and incubated with 1:200 mouse IgM anti-O4 antibody (R&D Systems, Madrid, Spain) in PBS with 10% goat serum overnight at 4 °C. After washing with PBS, slides were incubated with secondary antibody (anti-mouse IgG Alexa Fluor^®^ 488; Invitrogen, Madrid, Spain) 1:200, 2% goat serum and 1% Hoechst 33258 (Sigma Aldrich, Madrid, Spain) for nuclei counterstaining in PBS for 30 min at 37 °C. After washing with PBS, slides were mounted with Fluoromount-G™ Mounting Medium (Invitrogen, Madrid, Spain) and stored at 4 °C until image acquisition. Two images per neurosphere were taken with a BX61 microscope (Olympus, Tokyo, Japan) and analyzed with ImageJ 1.53a. The number of O4+ cells was manually counted and normalized by the number of nuclei. For the time-course assay OL differentiation was analyzed every 24 h over 5 consecutive days. Additionally, a maturation evaluation was performed using the same images: OLs were classified in different maturation stages according to their morphological appearance: immature, bipolar, mature, and myelinating (Figure 2D). The cell number of each maturation stage was normalized by the total number of O4+ cells. At least three independent experiments were performed for each endpoint. BMP7 [100 ng/mL] was used as positive control in every experiment.

#### 2.3.4. Cell Viability

Cell viability was assessed by using CellTiter-Blue^®^ cell viability assay (Promega, Madrid, Spain). This assay is based on the measurement of mitochondrial reductase activity of living cells by conversion of resazurin to the fluorescent product resorufin. After 2 h of incubation with the reagent (1:3 *v*/*v*), the medium was placed in a 96-well plate and read with FLUOstar Optima microplate reader. Cell viability was determined after 5 days of differentiation and for the time-course-assay every 24 h over 5 consecutive days. Neurospheres exposed to 10% DMSO (2 h) were used as lysis control in every experiment.

#### 2.3.5. qRT-PCR

After 5 days of differentiation, RNA was isolated, cDNA synthesized, and qRT-PCR performed. A detailed description of the method and primer sequences are given in SM2 and Table S2.

### 2.4. Statistics

Statistical analyses were performed using GraphPad Prism v9. Comparisons of two groups and time-course experiments were performed with a two-way ANOVA analysis. Significance over time was assessed by one-way ANOVA. Concentration-dependent effects were assessed by performing a one-way ANOVA. Post-hoc test Bonferroni’s multiple comparison test followed ANOVA analysis. The difference between SC and one sample was calculated with a two-tailed student’s *t*-test. The significance threshold was established at *p* < 0.05.

## 3. Results

### 3.1. IUGR Decreases OL Differentiation

Our previous study on the effects of IUGR in rabbit neurospheres already detected a significantly lower percentage of O4+ cells after 5 days in vitro [14]. This result was reproduced in the present study ( A and Figure S1) and further investigated to distinguish if this significantly lower percentage at 5 days in vitro was due to a decrease in differentiation or to an increase in cell death.

Neurospheres were obtained from 12 control and 10 IUGR PND0 pups with a significantly reduced birth weight compared to control (Control: 48.52 ± 1.93 g, IUGR: 31.72 ± 2.17 g, Table 1). In a time-course assay over 5 days (Figure 2B) in both, the control and IUGR groups, the percentage of O4+ cells increased significantly over time (Control *p* = 0.0006; IUGR *p* = 0.0013). However, there was no increase in % O4+ cells in the IUGR culture between days 3 and 4, and on days 4 and 5 the percentages were significantly lower in IUGR than in control neurospheres. The time-course experiment revealed that the differentiation rate in IUGR neurospheres is slower than in control (Figure 2B). This effect was not derived from cytotoxicity since cell viability remained comparable between groups at all time points (Figure 2C). There was also no decline in the % of O4+ cells in IUGR neurospheres over time indicating no specific death of this cell type (Figure 2B). During the 5 days of study, OLs underwent several maturation stages with increasing morphological complexity from immature appearance over bipolar and mature until they reached their myelinating postmitotic stage (Figure 2D,E). Over time, the immature stage decreased significantly while mature and myelinating stages increased significantly (Figure 2E). On the first day of differentiation, in control and IUGR cultures, the OL population was composed of 88–91% immature OLs (green) while on day 2 more OLs developed a bipolar (yellow, control 26.20%; IUGR 22.37%) or mature morphology (orange, control 13.41%; IUGR 16.87%). The number of mature OLs increased on day three and remained as the main OL population until day five (orange, control 54.42%; IUGR 54.38%). In the IUGR group, the myelinating stage was significantly lower on day four (red, control 11.17%; IUGR 4.40%; *p* = 0.0183) but increased on day five to reach a value comparable to control (red, control 10.16%; IUGR 10.74%) suggesting a delayed ability to mature (Figure 2E,F).

Besides the morphological appearance, the gene expression of the OL lineage maturation markers *Mbp* and *Mog* was analyzed on day 5 in control and IUGR neurospheres without exposure or after exposure to T3 [3 nM] as a positive control (Figure 2G), since T3 is known to increase the OL maturation in human and rat neurospheres [21]. The OL marker *Mbp* is expressed in mature and myelinating OLs, while *Mog* is expressed in the postmitotic state of myelinating OLs [35]. IUGR showed a mild downregulation of *Mbp* (0.71-fold) as well as *Mog* (0.70-fold) expression relative to the control. T3 significantly enhanced the expression of *Mbp* (control: 4.3-fold; IUGR: 2.4-fold) and *Mog* (control: 1.4-fold; IUGR: 1.3-fold) in control and IUGR neurospheres, as expected.

During the 5 days in differentiation culture, neural progenitor cells migrated out from the neurosphere core and differentiated while migrating (Figure 2H). In both the control and IUGR groups, the migration distance increased over the first three days and remained almost constant until day 5 (Figure 2I). Accordingly, the migration rate decreased over time and exhibited analogue dynamics between IUGR and control neurospheres over the 5 days (average migration rate: 11.3 µm/h (control); 12.0 µm/h (IUGR); Figure 2J).

With this first evaluation, we proved that IUGR significantly impairs OL differentiation in rabbit neurospheres and that IUGR neurospheres present a slower differentiation rate compared to controls, while migration rate and cell viability remain unaffected. A morphology and gene expression analysis indicated a mild delayed OL myelination due to IUGR.

### 3.2. In Vitro Testing of Potential Therapies

**Figure 3:**
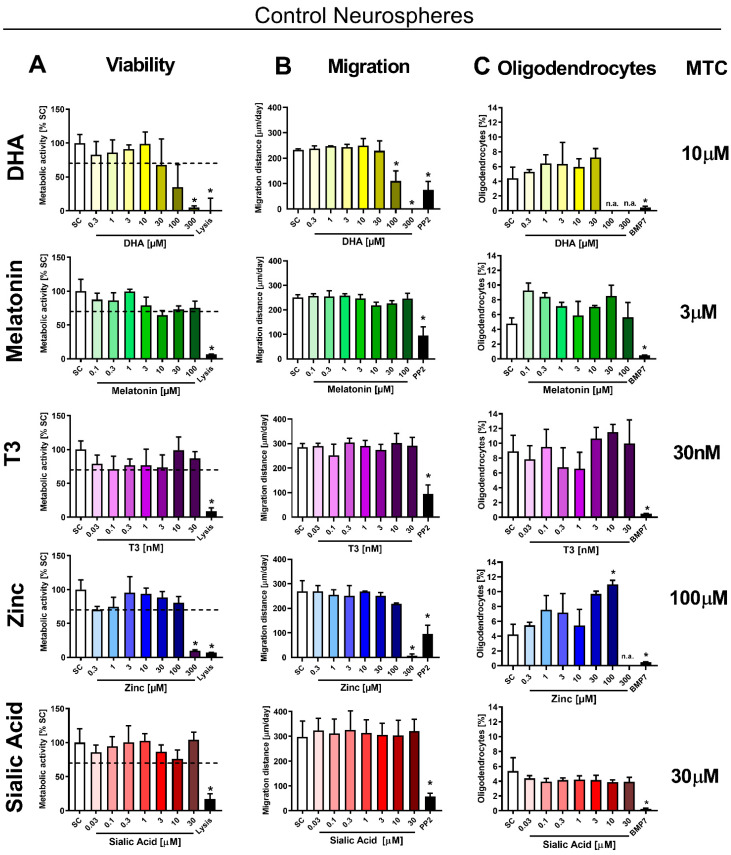
Maximum tolerated concentrations (MTCs) of potential therapies in control neurospheres. Rabbit neurospheres obtained from control pups were cultured for 3 or 5 days and tested for each endpoint with increasing concentrations of DHA, SA, MEL, zinc, T3, and an endpoint specific positive control. (**A**) Viability determined by metabolic activity after 5 days, positive control: lysis (10% DMSO), dotted line: 70 % of SC, (**B**) migration distance per day (mean ± SEM), positive control: PP2, (**C**) oligodendrocyte differentiation after 5 days (mean ± SEM), positive control: BMP7 [100 µM]. MTC: maximum tolerated concentration of each compound. All endpoints were evaluated in 5 neurospheres/condition in at least 3 independent experiments. n.a.: not analyzed. *: *p* < 0.05 vs. solvent control (SC).

Intending to foster the OL population under IUGR conditions, we tested five potential therapies in the neurosphere assay. The therapies were selected based on literature with preliminary evidence to prevent or revert the perinatal adverse results and neurological damage associated with IUGR: DHA, MEL, T3, zinc and LF as well as its main metabolite SA. LF, as a lactic compound, was not soluble in the medium (Figure S2), and thus SA was considered as a replacement candidate for in vitro experiments [34]. In a first approach, we determined the maximum tolerated concentration (MTC) of all potential therapies in control neurospheres (Figure 3). The criteria to set the MTC was viability higher than 70% of SC and no significant adverse effect in migration distance or OL differentiation.

The migration distance and OL differentiation were not specifically disturbed by any of the tested compounds in control neurospheres. A significant effect was only observed because of general cytotoxicity at high concentrations of DHA and zinc (100 and 300 µM DHA and 300 µM zinc; Figure 3A,B). DHA at 30 μM reduced the viability to 67.5 ± 38.4%, which was already below the acceptance limit. OL differentiation and migration were not significantly altered at concentrations below 30 µM (Figure 3C). Taking all three endpoints into account, MTC for DHA was established at 10 µM. MEL at 10 µM displayed reduced viability to 64.7 ± 6.8% of control value and no reduced OL differentiation or migration distance. In consequence, the MTC for MEL was set to 3 µM. The MTC of T3 was established to the highest tested concentration (30 nM) since viability, migration and OL differentiation was not significantly reduced at any tested concentration. 300 µM zinc significantly reduced the metabolic activity, and therefore the MTC was set to 100 µM. Here, in control neurospheres 100 µM zinc significantly increased the percentage of OL compared to SC (SC: 4.21%; 100 µM zinc 10.98%; *p*= 0.0238). SA did not reduce viability, migration, or OL differentiation, thus its MTC was established at the highest tested concentration (30 µM).

**Figure 4:**
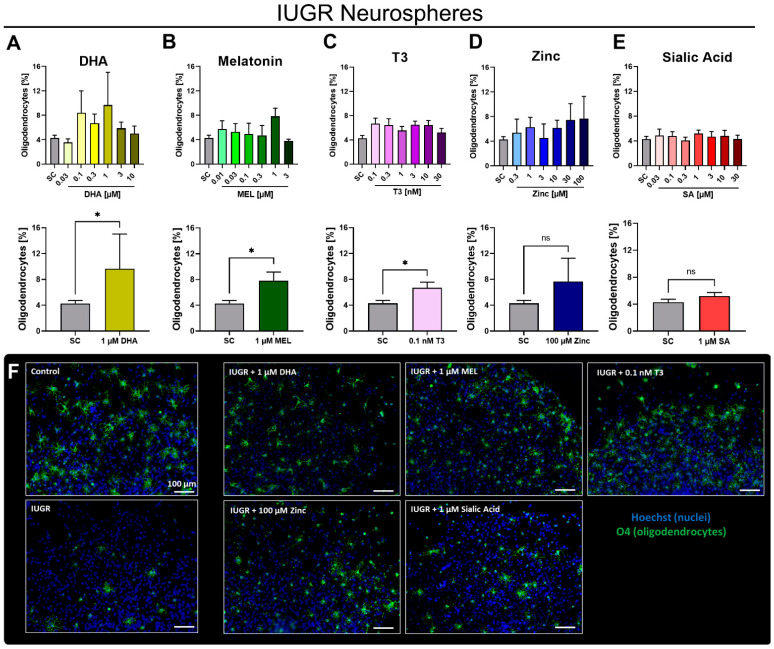
Effective concentrations of potential therapies in IUGR neurospheres. Rabbit neurospheres obtained from IUGR pups were cultured for 5 days and tested for oligodendrocyte percentage (mean ± SEM) with increasing concentrations (upper row) or the most effective concentration (lower row) of (**A**) DHA, (**B**) MEL, (**C**) zinc, (**D**) T3 and (**E**) SA. (**F**) Representative pictures of control and IUGR neurospheres; and of IUGR neurospheres exposed to the most effective concentration of DHA, MEL, T3, zinc and SA. Oligodendrocyte marker O4 (green) and Hoechst 33,258 (blue), Scale bar = 100 µm. Analysis was evaluated in 5 neurospheres/condition in at least 3 independent experiments. ns: not significant, *: *p* < 0.05 vs. solvent control (SC).

The main interest was to find a concentration of the tested therapies which enhanced the OL differentiation of IUGR neurospheres to control neurosphere levels. Migration distance and viability assays were simultaneously performed to overcome the adverse effects of the therapies in these endpoints (Figure S3). Rabbit neurospheres from IUGR pups were exposed to the potential therapies with increasing concentrations up to their respective MTC (Figure 4A).

None of the potential therapies produced a significant concentration-dependent monotonic response in IUGR neurospheres. However, some of them induced non-monotonic responses with high increases in oligodendrocyte differentiation at specific concentrations. Exposure to 1 µM DHA increased the OL differentiation to its maximum (9.7 ± 5.4%) and showed a significant increase by comparison to the SC. Therefore, 1 µM DHA was considered as its most effective concentration in vitro (Figure 4A).

Notably, 1 µM MEL significantly increased the OL differentiation of NPCs obtained from IUGR pups (7.8 ± 1.3%) and was set as its most effective concentration (Figure 4B). T3 showed a significant increase of the OL population in IUGR neurospheres with the lowest tested concentration (0.1 nM, 6.7 ± 0.9%). Thus, 0.1 nM T3 was determined as the most effective concentration (Figure 4C). Conversely, none of the tested zinc concentrations (not even the maximum tested concentration of zinc: 100 µM, 7.7 ± 3.6%) significantly increased the percentage of O4+ cells compared to the differentiation in IUGR neurospheres. Consequently, we did not consider zinc as a promising therapy to reduce adverse outcomes induced by IUGR (Figure 4D). Additionally, SA did not increase the OL population in IUGR neurospheres at any tested concentration. Figure 4F displays representative pictures of control and IUGR neurospheres, as well as IUGR neurospheres exposed to the most effective/best concentration of DHA, MEL, T3, zinc and SA. Based on these results, we can conclude that the compounds DHA, MEL and T3 are the most promising ones due to their stimulating effect in OL differentiation.

### 3.3. In Vivo Administration of Selected Therapies

**Figure 5:**
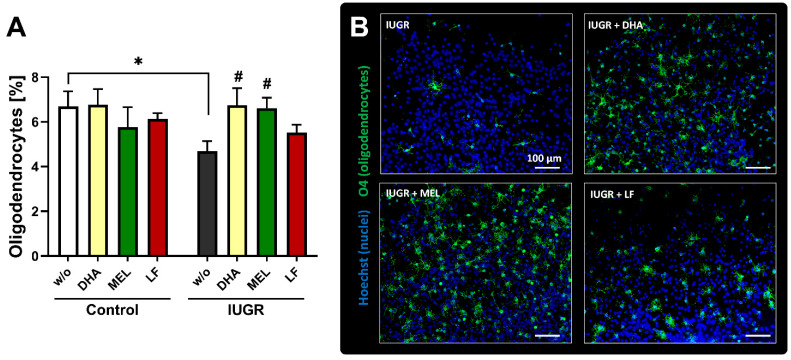
In vivo administration of selected therapies. Oligodendrocyte differentiation. Pregnant rabbits were administered to either MEL (10 mg /kg BW/day), DHA (37 mg/kg BW/day) or LF (166 mg/kg BW/day) at the day of IUGR induction until caesarean section. w/o = rabbit does without administered therapy. (**A**) Neurospheres obtained from control and IUGR pups were tested for % oligodendrocyte differentiation. (**B**) Representative pictures of IUGR neurospheres w/o and with administered therapies; Oligodendrocyte marker O4 (green) and Hoechst 33258 (blue), Scale bars = 100 µm. Analysis was evaluated in 5 neurospheres/condition in at least 3 independent experiments. Mean ± SEM; * *p* < 0.05 vs. w/o control, # *p* < 0.05 vs. w/o IUGR.

To confirm the in vivo relevance of these results, we selected DHA, MEL and LF for daily administration during pregnancy after IUGR induction until C-section. In this study, T3 was not prioritized due to the higher difficulties this therapy would present in the future when transferred to the clinical field [36]. Although SA, the main metabolite of LF, was not effective in vitro (see Section 3.2), LF was still selected for in vivo administration, because a negative effect of the metabolite in vitro does not exclude a positive effect of the parent compound in vivo. The birth weight of IUGR pups from all treatment groups was significantly reduced compared to control pups in the untreated groups (Table 1), implying that the therapies did not interfere with the birth weight. This means that if a protective effect were detected in one therapy, it could not be assigned to a mere change in growth. Indeed, neurospheres from IUGR pups delivered from rabbits dosed with DHA presented a significantly increased percentage of OLs up to the control value (Figure 5). Whereas the OL population in control neurospheres after dosing with DHA remained on the control level. The cellular metabolic activity and migration were not diminished after DHA treatment (Figure S4). Moreover, the prenatal administration of MEL also significantly promoted the OL differentiation and increased the number of O4+ cells in IUGR cases to the control value (Figure 5). Besides, the viability did not differ significantly from the controls (Figure S4). Finally, our results showed that LF administered to the rabbit carrying control and IUGR pups could not revert the reduced OL population in IUGR cases (Figure 5). In this case, cell viability and migration were also not disturbed (Figure S4). From these results, DHA and MEL were identified as the best therapies among the tested ones, but no difference between them could be detected. The lack of adverse effects of these therapies in migration or viability is a preliminary information on the safety of these potential therapies during neurodevelopment.

## 4. Discussion

In this study, we identified DHA and MEL as the most effective neuroprotective therapies for IUGR-induced oligodendrogenesis alterations. These two therapies were selected among five candidates using an in vitro approach, the rabbit neurosphere assay, and confirmed in this model after in vivo treatment.

Neurospheres have been used for many years as a model to study central nervous disorders including Alzheimer’s, Parkinson’s, demyelinating diseases, epilepsy and glioma [19], but it was not until recently that a rabbit and a rabbit IUGR neurosphere model were established [14], opening the door for investigations of the effects of IUGR on cell functions which are characteristic of the developing brain, and to test potential neuroprotective therapies in a more time- and cost-efficient way than traditional in vivo studies. The rabbit species was chosen because its brain development occurs largely perinatally, like in humans [14,37,38], whereas the rodent brain develops mainly during the prenatal phase [39]. In our previous studies with the rabbit neurosphere assay, we proved the ability of the model to identify the developmental neurotoxicity of known neurotoxicants like MeHgCl, and by using the IUGR rabbit neurospheres, we identified an adverse impact of IUGR on oligodendrogenesis. In the present study, we further characterized this oligodendrogenesis impairment and have applied the IUGR neurosphere model for drug screening for the first time.

The already identified oligodendrogenesis impairment consisted of a lower percentage of oligodendrocytes after 5 days in vitro. As such, we have determined that the main insult emerges already in pre-myelinating O4+ OLs. Our results reveal a significantly lower percentage of O4+ cells at day 4 in culture and a slower OL differentiation rate in IUGR neurospheres compared to control without an increase in specific cell death. Regarding myelination, our findings indicate that only at day 4 is myelination significantly reduced in IUGR neurospheres; however, this difference is only present for a short time in the culture, since 24 h later, the percentage of myelinating OL increases to the control level. This 24 h delay in myelination is in accordance with other studies of induced perinatal hypoxia-ischemia in rodents, indicating a failure of maturation rate in oligodendrocyte progenitor cells (OPC) or pre-myelinating OLs [40,41,42]. At the final time-point (day 5), maturation was assessed in two ways, i.e., OL morphological analysis and gene expression analysis of *Mbp* and *Mog.* Both methods delivered the same result, with no significant differences in myelination between control and IUGR. Therefore, in this case, these are comparable alternatives with different advantages, i.e., morphological evaluations with no extra cost but increased duration, and gene expression analyses with extra cost but reduced duration. The oligodendrogenesis alterations we found correlate very well with clinical findings indicating that brain damage in IUGR infants is related to white matter injury in its diffuse form, as this injury is associated with a selective vulnerability of the OL lineage [40,43]. Back et al. described it as a differentiation failure of newly generated pre-OLs, because these are especially susceptible to free radicals while later OL stages appear to be more resistant [40]. Other studies in primary rat OPC cultures with induced oxidative stress found that the expression of genes stimulating OL differentiation was downregulated, while the expression of genes inhibiting OL differentiation was upregulated [43]. Based on our results, potential neuroprotective therapies were devised to prevent this selective OL lower differentiation and not focus on increasing the OL myelinating status, since this effect disappeared spontaneously after 24 h in our culture.

Potential neuroprotective therapies are required to be effective after a short treatment period, since late-onset IUGR is the most frequent IUGR-type, with an incidence of 70–80% of IUGR cases [44]. In these cases, there is often only a small time window between identification of the risk and intervention leading to a reduced opportunity for prevention/correction [45]. Nowadays the common clinical approach is induced delivery because no effective therapy for IUGR has been established to date [45,46]. Our neurosphere model reflects an IUGR condition induced at a late stage of pregnancy [14,15], and therefore, is a useful preclinical technique for the screening of new antenatal neuroprotective therapies. Based on literature research, we selected five potential therapies to overcome IUGR-induced brain insult (DHA, MEL, T3, Zinc, and LF), and for all of them, we characterized the MTC in rabbit neurospheres in vitro. Among these five potential therapies, DHA (1 µM) and MEL (1 µM) could revert the reduced level of O4+ cells in vitro and were able to prevent pathological effects secondary to IUGR by administration during rabbit’s pregnancy (37 mg/kg BW/day and 10 mg/kg BW/day, respectively).

DHA, an omega-3 fatty acid, is delivered maternally through uteroplacental circulation and is a key component of brain membrane phospholipids [23,24]. It is critical to fetal central nervous system growth and development; however, the majority of pregnant women do not consume an adequate amount of omega-3 fatty acids on a regular basis [22]. Maternal DHA supplementation in a rat model prevented neonatal brain injury by reducing oxidative stress and apoptotic neuronal death [47]. Other studies support the hypothesis that DHA enhances the differentiation of OL progenitors into mature OL in demyelinating diseases [48]. Several clinical studies have already been performed administering DHA in different forms to pregnant women, aiming to prevent other disorders, e.g., infant cardiac outcomes or depressive symptoms during pregnancy or postpartum, with positive and negative outcomes being reported [49,50]. In either case, no general safety concerns have been identified. Our results, together with this evidence, strongly support the proposal of a clinical trial of DHA administration to pregnant women carrying fetuses with IUGR to prevent white matter IUGR-induced alterations.

MEL is produced in the placenta and ovary, where it works as a free radical scavenger and potent antioxidant and has an essential function in placental homeostasis and fetal maturation [51]. Studies about pregnancies complicated with placental insufficiency revealed a significantly reduced MEL level in maternal blood [52] and significantly reduced expression of MEL receptors in the placenta [53]. In animal studies, MEL improved neurological outcomes in an ovine model [25,54] and a rat model of white matter damage [55,56]. To date, only one pilot clinical trial has been undertaken of oral MEL administration to women with IUGR [25]. Our results reinforce the evidence that MEL is a promising antenatal neuroprotective therapy due to its promoting effect in fetal pre-OL differentiation.

In our study, an in vitro exposure to T3 significantly increased the OL differentiation and the expression of the myelination marker *Mbp* and *Mog* in IUGR neurospheres. This effect is in accordance with increased O4+ cells and myelination in rodent and human neurospheres after T3 exposure [21,57]. However, due to the good results obtained with DHA and MEL, T3 was not prioritized in our study for in vivo maternal administration due to possible complications altering the maternal T3 level, and thus, to expected difficulties in the translation to the clinical field [36,58]. Clinical findings of reduced circulating thyroid hormone concentrations in severe IUGR fetuses prompted further investigations of the potential therapeutic role of peripartum thyroid hormone treatment; however, the reader is referred to the recent review of LaFranchi (2021) [59], which summarizes the mixed results of clinical trials and comments on unresolved questions and the main current areas of controversy surrounding this treatment. Surprisingly, exposure to zinc did not significantly increase the percentage of O4+ cells in IUGR neurospheres (Figure 3). This treatment was discarded for further investigations in the IUGR model, but these results could be useful for other researchers aiming to increase the percentage of O4+ cells in other disease models. Sialic acid, the main metabolite of LF, did not alter OL differentiation in vitro, nor did LF (166 mg/kg BW/day) after maternal treatment in vivo in our study. LF, an iron-binding glycoprotein with antioxidant and anti-inflammatory properties, reportedly supports the growth of neurons, maintains neuronal integrity, and increases neuronal density during brain development [34]. Administered postnatally to rat pups with cerebral hypoxic-ischemic injury, it shows neuroprotective effects on brain metabolism, and cerebral gray and white matter recovery [60]. This notwithstanding, in our model, no protective effect was observed after in vitro or in vivo treatment, perhaps due to the shorter treatment period, to the earlier evaluation time-point or the different endpoints measured.

Overall, it is remarkable that the in vitro rabbit neurosphere assay recently established by our group [14] predicted, in all of the studied endpoints, the in vivo outcome of the tested potential neuroprotective candidates, for both positive and negative effects. The good agreement of the in vitro and in vivo treatments increases the reliability of the model and supports its use for further drug screening. In future studies, it will be important to measure the impact and safety of the tested therapies not only in oligodendrogenesis, but also on neuronal differentiation in IUGR and control neurospheres, as well as their safety in terms of general developmental parameters.

## 5. Conclusions

Using the rabbit IUGR neurosphere model, we demonstrated an adverse impact of IUGR on the differentiation rate of pre-myelinating O4+ OL and identified two therapies, i.e., DHA, and MEL, which are able to revert the reduced OL percentage in rabbits in vitro and prevent the impairment in vivo by maternal administration during pregnancy.

## Figures and Tables

**Table 1:** Number and birth weight of PND0 rabbit pups included in the study.

Treatment	Dose (mg/kg bw/day)	Number of Control Pups	Birth Weight [g] ± SEM	Number of IUGR Pups	Birth Weight [g] ± SEM
	**Rabbit Doe**	**Control Pups**		**IUGR Pups**	
**w/o**	-	12	48.52 ± 1.93	10	31.72 ± 2.17 *
**DHA**	37	2	57.05 ± 3.90	2	34.76 ± 5.20 *
**MEL**	10	2	52.01 ± 9.38	2	27.94 ± 2.52 *
**LF**	166	2	59.72 ± 1.57	2	37.86 ± 3.71 *

From one rabbit pup’s whole brain, at least four independent experiments were performed. The rabbit pup’s sex is not visible at PND0 and was not determined. The dose administered to the pregnant rabbit is indicated, w/o: without treatment, bw: bodyweight, *: *p* < 0.05 vs. corresponding control birth weight.

**Table 2:** In vitro testing concentrations of potential therapies.

Compound (Synonym)	CAS Number	Solubility	Concentration In Vitro	MTC
**DHA**	6217-54-5	300 µM (DMSO)	300–100–30–10–3–1–0.3 µM	10 µM
**MEL**	73-31-4	100 µM (DMSO)	100–30–10–3–1–0.3–0.1 µM	3 µM
**T3**	55-06-1	30 nM (HCl/EtOH)	30–10–3–1–0.3–0.1–0.03 nM	30 nM
**Zinc**	7440-66-6	300 µM (H_2_O)	300–100–30–10–3–1–0.3 µM	100 µM
**LF**	339615-76-8	10 mg/mL (H_2_O)	30–10–3–1–0.3–0.1–0.03 µM	30 µM
**SA**	131-48-6	30 µM (DMSO)	30–10–3–1–0.3–0.1–0.03 µM	30 µM

The tested compounds, the concentration range used in vitro based on their solubility and the resulting maximum tolerated concentration (MTC) is described. The maximum solvent percentage submitted was 0.1% (*v*/*v*).

## Data Availability

The data that support the findings of this study are available from the corresponding author upon reasonable request.

## Supplementary Materials

The following supporting information can be downloaded at: https://www.mdpi.com/article/10.3390/biomedicines10051205/s1, [61,62,63,64,65]. Figure S1: Viability of control and IUGR neurospheres; Figure S2: Maximum tolerated concentrations of lactoferrin; Figure S3: Viability and migration of IUGR neurospheres; Figure S4: In vivo administration of selected therapies. Viability and Migration distance; Table S1: Summary of the literature supporting the selection of the five potential therapies; Table S2: Primer sequences 5′–3′ designed with NCBI Blast Primer design; SM1: In vivo treatment calculations and supplier; SM2: qRT-PCR.
