# Supplementary material for: Docosahexaenoic Acid and Melatonin Prevent Impaired Oligodendrogenesis Induced by Intrauterine Growth Restriction (IUGR)
Source: Biomedicines. 2022 May 23;10(5):1205. doi: 10.3390/biomedicines10051205 (PMC9138514; doi:10.3390/biomedicines10051205)
Supplement: Supplementary file 1 [file biomedicines-10-01205-s001.zip › biomedicines-1722314-supplementary.pdf]

biomedicines

MDPI

Supplementary Materials:

Table S1. Summary of the literature supporting the selection of the five potential therapies.

Potential Therapy	Study type	Species	Previous effect described	References
DHA	review	Human	- Omega-3 fatty acids are critical for fetal neurodevelopment and may be important for the timing of gestation and birth weight.<br>- For pregnant women to obtain adequate omega-3 fatty acids, a variety of sources should be consumed.	[22]
	Distribution and human placental transfer of ^{13}C-DHA 12 h after oral application.	Human	- Placenta-maternal plasma ratios and fetal-maternal plasma ratios for ^{13}C-DHA were significantly higher than those for any other fatty acids.<br>- ^{13}C-DHA is predominantly esterified into phospholipids and triglycerides in maternal plasma, which may facilitate its placental uptake and transfer.	[23]
	review	Cell and animal studies	- Its accumulation in the fetal brain takes place mainly during the last trimester of pregnancy and continues at very high rates up to the end of the second year of life.<br>- DHA intake may contribute to optimal conditions for brain development.<br>- DHA in related to brain function for neuronal cell growth, differentiation and to neuronal signaling	[24]
MEL	In vivo:<br>- IUGR induction at 0.7 gestation in sheep with or without MEL intravenous infusion until term.<br>- Neonatal behavioral assessment to examine neurological function and at 24 hr after birth the brain was collected for the examination of neuropathology.	Sheep	- IUGR lambs took significantly longer to achieve normal neonatal lamb behaviors. IUGR brains showed widespread cellular and axonal lipid peroxidation, and white matter hypomylination and axonal damage.<br>- Maternal melatonin administration ameliorated oxidative stress, normalized myelination and rescued axonopathy within IUGR lamb brains	[25]
	Pilot clinical trial of oral melatonin administration to women with an IUGR fetus.	Human	- Maternal melatonin significantly reduced oxidative stress, as evidenced by reduced malondialdehyde levels, in the IUGR + MEL placenta compared to IUGR alone.	[25]
	In vivo:<br>IUGR induction in pregnant rats,<br>intra-peritoneal injection of melatonin from birth to PND3.	Rat	- At PND14, melatonin prevented white matter myelination defects with an increased number of mature oligodendrocytes<br>- Melatonin was not found to be associated with an increased density of total oligodendrocytes (Olig2-immunoreactive), suggesting that melatonin is able to promote oligodendrocyte maturation but not proliferation.	[55]

			- These effects appear to be melatonin-receptor dependent and were reproduced in vitro.	
Zinc	review	Human, Mouse	- Impaired activation of the extracellular signal-regulated kinases (ERK1/2) contributes to the disruptions in neurodevelopment associated with zinc deficiency. These kinases are implicated in major events of brain development, including proliferation of progenitor cells, neuronal migration, differentiation, and apoptotic cell death.	[26]
	review		- A decrease in neuronal zinc causes cell cycle arrest, which in part involves a deregulation of select signals (ERK1/2, p53, and NF-jB).<br>- Zinc deficiency induces apoptotic neuronal death through the intrinsic (mitochondrial) pathway<br>- Zinc deficiency induces alterations in the finely tuned processes of neurogenesis, neuronal migration, differentiation, and apoptosis, which involve the developmental shaping of the nervous system.	[27]
	In vivo:<br>Effect of zinc on brain development was investigated during the lactation in undernourished mouse pups.	Mouse	- Significant increase in the volume of CA1 neuronal cells in undernourished control mice, which was not seen in mice receiving zinc<br>- Zinc protects against malnutrition-induced brain developmental impairments.	[28]
	Randomized Controlled Trial:<br>To assess the effect of zinc supplementation on neuro-development and growth of preterm neonates.	Human	- Zinc supplementation till 3 month corrected age in preterm breastfed infants improves alertness and attention pattern; and decreases signs of hyperexcitability, and proportion with abnormal reflexes.	[29]
T3	In vitro rat neurospheres: derived from the subventricular zone of adult rats	Rat	- T3 reduced the proliferation rate of neurospheres when cultured in the presence of mitogens, shifting towards oligodendroglial lineage as indicated by increased expression of olig-1, and also favoring oligodendrocyte maturation.	[30]
	Review		- Several hormones including thyroid hormones, carefully regulate the growth of the fetus and its metabolism during pregnancy by controlling the supply of nutrients crossing the placenta.<br>- Thyroid hormones are essential for fetal brain development and maturation.	[31]
	Review		- Thyroid hormones, especially T3, are essential for brain development through specific time windows influencing neurogenesis, neuronal migration, neuronal and glial cell differentiation, myelination, and synaptogenesis.	[32]

LF	In vivo: Moderate brain hypoxia-ischemia was induced in the PND3 rat brain (24-28 weeks human equivalent).	Rat	- LF given through lactation to rat pups with cerebral hypoxia-ischemia injury shows neuroprotective effects on brain metabolism, and cerebral gray and white matter recovery.	[33]
	Review		- LF supports neuronal outgrowth and synaptic connectivity during fetal brain development	[34]

SM1: ***In vivo* treatment calculations and supplier**

Specific doses for each treatment were calculated extrapolating the most effective concentrations *in vitro* determined in section 3.2 to *in vivo* doses according to previous pharmacokinetic studies specific for each treatment. The dose administered *in vivo* for DHA (37 mg/kg bw/day) was extrapolated from human pharmacokinetic (PK) parameters [61], for MEL (10 mg/kg bw/day) from human PK data reviewed by Harpsøe et al 2015 [62] as well as data from pregnant sheep [63]. Since for LF a most effective concentration *in vitro* could not be determined (see section 3.2), the dose (166 mg/kg bw/day) was selected taking into account previous neuroprotective effects described *in vivo* in rats [60] and adjusted to rabbit species using interspecies translation equations described elsewhere [64,65]. DHA was obtained from Rendon Europe Laboratories and presented in 3 g 100 % pure powder from Microalga oil Schizochytrium sp. MEL was supplied in a suspension for oral administration [20 mg/mL] containing sorbitol solution 70 %, Sodium benzoate, Xanthan gum, and double-distilled water prepared at the Pharmacy service of Hospital Sant Joan de Déu Barcelona. Bovine LF containing a low concentration of iron (9.2 mg of iron per 100 g of protein) was obtained from Farmalabor.

SM2: qRT-PCR

After 10-12 days of proliferation, IUGR and control pup's derived neurospheres were chopped to 0.1 mm pieces and resuspended in N2 differentiation medium. 700-800 pieces were placed in a PDL/Laminin coated well on a 24-Well-plate containing 1 mL differentiation medium. Neurosphere pieces were cultivated for 5 days in triplicates either untreated or exposed to 3 nM T3. Half of the medium was changed to fresh medium after 72 h of cultivation. After 5 days RNA was isolated (RNeasy Mini Kit, Qiagen, Hilden, Germany) and cDNA was transcribed (Quantitect Reverse Transcription Kit, Qiagen) according to the manufacturer instructions. Primer sequences were designed using NCBI Primer-BLAST (Table S1). qRT-PCR was performed using QuantiFast SYBR Green PCR Kit (Qiagen) and the AriaMx Real-Time PCR instrument with the software Agilent Aria Software v1.6. The thermal profile included a hot start for 7 min at 95°C, 47 amplification cycles comprising 10 min at 95°C for denaturation, 35 min at 60°C for annealing and 20 min at 72°C for extension, followed by a melting step for the dissociation of PCR products for 30 min at 75°C and 30 min at 99°C. Gene expression was evaluated by using the cycle threshold (Ct) value from each sample. Fold change expression of the gene of interest (GOI) was assessed by the ΔΔCt method: The ΔCt between the Ct(GOI) and the corresponding Ct(β-actin) was calculated for each sample. The mean of the ΔCt values of the solvent control triplicates was assessed. The ΔΔCt was equal to the subtraction of the sample ΔCt and the mean of the solvent control ΔCt value followed by 2^-ΔΔCt. Mean and SEM of the qRT-PCR analysis was calculated from at least three independent experiments.

Table S2. Primer sequences 5′ – 3′ designed with NCBI Blast Primer design.

	Species	Forward primer	Reverse primer
Myelin basic protein (Mbp)	rabbit	CAAGGAAAGGGGAGAG GAAC	GAAGAGCCTGGAGAGTG TGC
Myelin oligodendrocyte glycoprotein (Mog)	rabbit	GGAGCTCTTGCTGAAAC ACC	CTTCAGGAACAAGGGCA GAG
β-actin	rabbit	TCCCTGGAGAAGAGCTA CGA	GTACAGGTCCTTGCGGAT GT

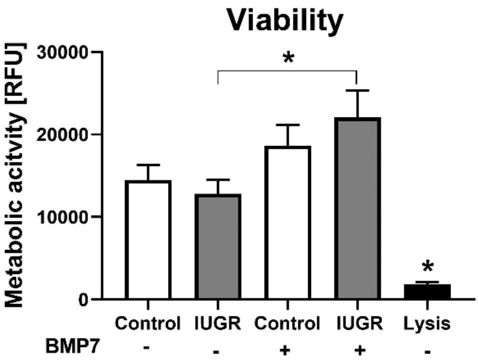

Figure S1. Viability of control and IUGR neurospheres. Rabbit neurospheres obtained from control and IUGR pups were analyzed for cell viability determined by metabolic activity [RFU] after 5 days of cultivation, positive control of oligodendrocyte differentiation BMP7 [100 ng/mL], lysis control (10% DMSO). Metabolic activity was evaluated in 5 neurospheres/condition in 15 independent experiments. Mean ± SEM; *: p<0.05 vs. control.

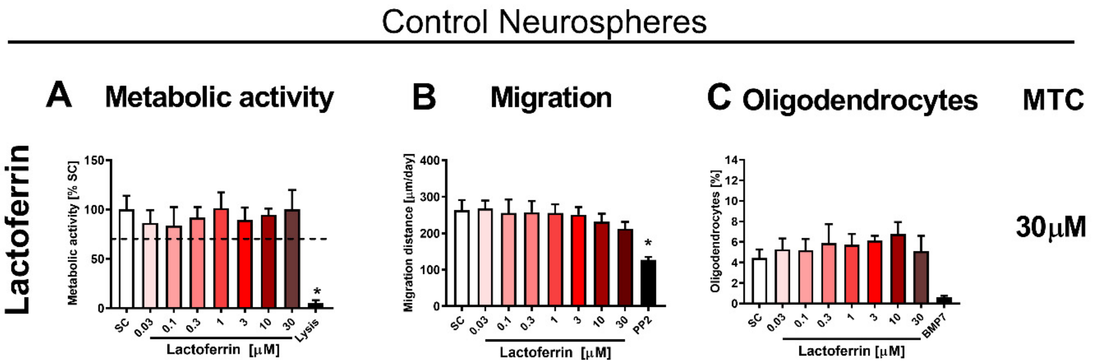

Figure S2. Maximum tolerated concentrations of lactoferrin. Rabbit neurospheres obtained from control pups were cultured for 3 or 5 days and tested for each endpoint with increasing concentrations of LF and an endpoint-specific positive control. (A) Viability analysis after 5 days, positive control: lysis (10% DMSO), dotted line: 70% metabolic activity, (B) migration distance per day (mean ± SEM), positive control: PP2 [10 µM], (C) oligodendrocyte differentiation after 5 days (mean ± SEM), positive control: BMP7 [100 ng/mL]. MTC: maximum tolerated concentration. All endpoints were evaluated in 5 neurospheres/condition in at least 3 independent experiments. *: p<0.05 vs. solvent control (SC).

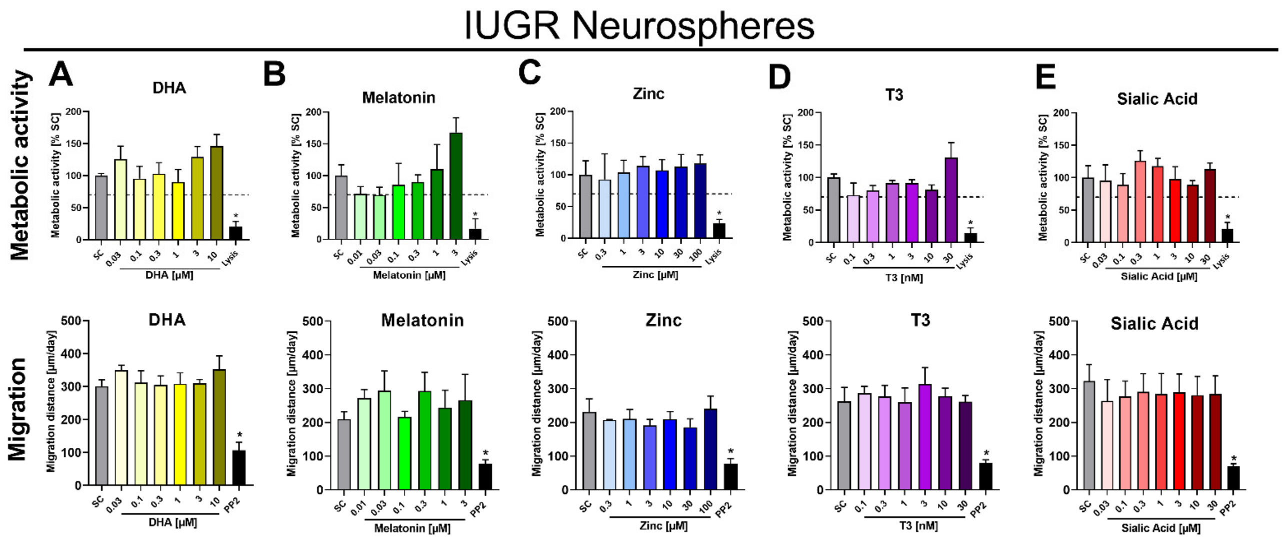

Figure S3. Viability and migration of IUGR neurospheres. Rabbit neurospheres obtained from IUGR pups were cultured for 5 days and tested for viability and migration distance (mean ± SEM) after exposure to increasing concentrations of (A) DHA, (B) melatonin, (C) zinc, (D) T3 and (E) sialic acid. Viability was determined by metabolic activity: analysis after 5 days, positive control: lysis (10% DMSO), dotted line: 70% metabolic activity. Migration distance per day, positive control: PP2 [10 µM]. Analysis was evaluated in 5 neurospheres/condition in at least 3 independent experiments. *: p<0.05 vs. solvent control (SC).

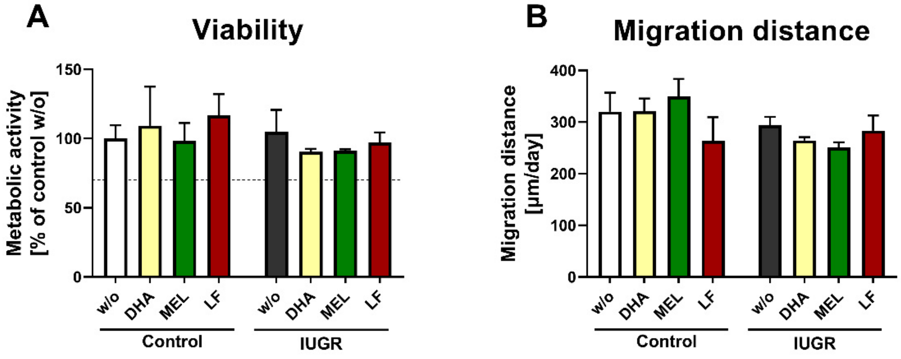

Figure S4. ***In vivo*** **administration of selected therapies. Viability and Migration distance.** Pregnant rabbits were not administrated (w/o) or either to MEL (10 mg /kg BW/day), DHA (37 mg/kg BW/day) or LF (166 mg/kg BW/day) at the day of IUGR induction until C-section. Neurospheres obtained from control and IUGR pups were tested for (A) viability determined by metabolic activity; black dotted line: 70% metabolic activity and (B) migration distance per day [µm]. Mean ± SEM.
